# A deep learning approach to median nerve evaluation in ultrasound images of carpal tunnel inlet

**DOI:** 10.1007/s11517-022-02662-5

**Published:** 2022-09-24

**Authors:** Mariachiara Di Cosmo, Maria Chiara Fiorentino, Francesca Pia Villani, Emanuele Frontoni, Gianluca Smerilli, Emilio Filippucci, Sara Moccia

**Affiliations:** 1 Department of Information Engineering, Università Politecnica delle Marche, Via Brecce Bianche 12, 60131 Ancona, AN Italy; 2 Department of Humanities, Università di Macerata, Macerata, Italy; 3 Department of Political Sciences, Communication and International Relations, Università di Macerata, Macerata, Italy; 4 Rheumatology Unit, Department of Clinical and Molecular Sciences, Università Politecnica delle Marche, “Carlo Urbani” Hospital, Ancona, Italy; 5 The BioRobotics Institute, Department of Excellence in Robotics and AI, Scuola Superiore Sant’Anna, Pisa, Italy

**Keywords:** Carpal tunnel syndrome, Deep learning, Median nerve, Segmentation, Ultrasound imaging

## Abstract

Ultrasound (US) imaging is recognized as a useful support for Carpal Tunnel Syndrome (CTS) assessment through the evaluation of median nerve morphology. However, US is still far to be systematically adopted to evaluate this common entrapment neuropathy, due to US intrinsic challenges, such as its operator dependency and the lack of standard protocols. To support sonographers, the present study proposes a fully-automatic deep learning approach to median nerve segmentation from US images. We collected and annotated a dataset of 246 images acquired in clinical practice involving 103 rheumatic patients, regardless of anatomical variants (bifid nerve, closed vessels). We developed a Mask R-CNN with two additional transposed layers at segmentation head to accurately segment the median nerve directly on transverse US images. We calculated the cross-sectional area (CSA) of the predicted median nerve. Proposed model achieved good performances both in median nerve detection and segmentation: Precision (*Prec*), Recall (*Rec*), Mean Average Precision (*mAP*) and Dice Similarity Coefficient (*DSC*) values are 0.916 ± 0.245, 0.938 ± 0.233, 0.936 ± 0.235 and 0.868 ± 0.201, respectively. The CSA values measured on true positive predictions were comparable with the sonographer manual measurements with a mean absolute error (*MAE*) of 0.918 *mm*^2^. Experimental results showed the potential of proposed model, which identified and segmented the median nerve section in normal anatomy images, while still struggling when dealing with infrequent anatomical variants. Future research will expand the dataset including a wider spectrum of normal anatomy and pathology to support sonographers in daily practice.

**Graphical abstract:**

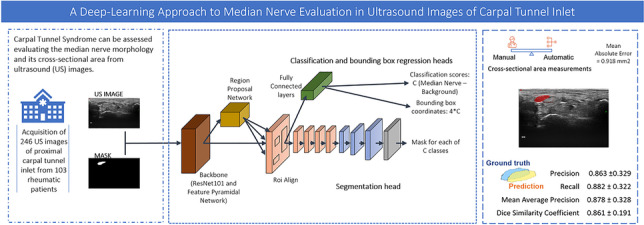

## Introduction

Carpal tunnel syndrome (CTS) accounts for 90% of peripheral entrapment neuropathies, affecting up to 5% of the general population [29]. This condition occurs when the median nerve is compressed at the wrist as it passes through a narrow osteofibrous canal along with the nine finger flexor tendons [20]. The median nerve stretches, compresses and translates in response to upper extremity motion, but in patients with CTS its mobility is restricted, which indicates nerve dysfunction [20].

Traditionally, the diagnosis of CTS relies on clinical history and physical examination [25], sometimes investigated further with electrodiagnostic tests, sensitive in examining nerve conduction and eventual damages [20]. Aside from electrodiagnosis, which is expensive, time-consuming and presents limited ability to predict CTS severity or intervention outcome [27], ultrasound (US) imaging can also be used. In assessing CTS, US allows to detect structural anomalies through the direct visualization of the nerve, its position and morphology: in fact, altered shape of the median nerve due to the compression of the surrounding nonrigid structures is expected in CTS patients [29].

Among the US parameters which can be evaluated from the carpal tunnel, the most common and reliable is the cross-sectional area (CSA) of the median nerve measured at the proximal carpal tunnel. However, the CSA measurements are currently performed on US relying on a hand tracing method, and their cutoff values for CTS diagnosis vary widely, ranging from 9 to 14 *mm*^2^ [29].

**Fig. 1:**
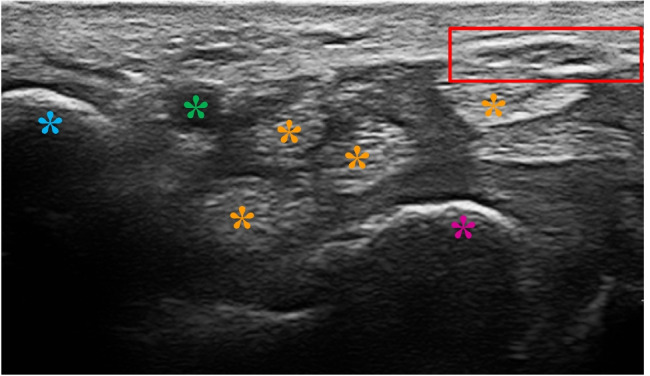
US transverse scan sample acquired at the proximal carpal tunnel inlet. A red box includes the median nerve section; asterisks of different colors mark other relevant structures: pisiform bone profile in blue, semilunar bone profile in purple, ulnar artery in green, digital flexor tendons in orange

US imaging presents unique challenges to be faced: it is highly dependent on sonographer’s experience, and subjected to high inter- and intra-observer variability across different manufacturers’ US systems. Moreover, US images can be subjected to low imaging quality, intensity inhomogeneities, presence of shadows and high noise level. In addition, in carpal tunnel imaging the median nerve identification is made harder by the presence of many rounded structures, such as the wrist bones, transverse carpal ligament and digital flexor tendons, and by nerve morphological variations in relation with disease severity, with other concomitant pathologies and also with height, sex, weight and age of the subjects [29]. A sample of carpal tunnel US image is shown in Fig. 1.

To address these challenges, the development of advanced automatic US image analysis methods is essential to make US a more objective and accurate support tool for CTS assessment. In this respect, Deep Learning (DL), which is a branch of machine learning, has already shown its huge potential for medical US analysis [22]. At present, multiple types of deep networks, especially Convolutional Neural Networks (CNN), have been successfully involved in various US images tasks, such as lesion/nodule classification, object detection and anatomical structure segmentation [22], thus implying DL potentiality to improve and standardize even CTS diagnosis through an automatic median nerve section identification.

Embracing this idea that DL may provide a reliable support to sonographers, we propose the present study in continuity with a previous preliminary work in [4]. While deepening our DL approach, we extended our dataset including 103 patients keeping into account anatomical variants occurrence. The contributions of this paper can be summarized as follows:

1. Development of an end-to-end CNN, i.e., a Mask R-CNN [13], for localization and segmentation of the median nerve at the inlet of the proximal carpal tunnel, further improved by the insertion of two additional transposed layers at segmentation head.
2. A comprehensive study conducted on transverse US images acquired in daily clinical practice.
3. Evaluation of CSA measurement based on the median nerve section segmented by the algorithm in comparison with manual tracing of nerve boundary performed by expert sonographers.

A more medical perspective and an analysis of the clinical implication of this work is reported in [26].

### Related work

Several studies faced the median nerve segmentation problem from US imaging involving model-based approaches. In [10], the phase-based probabilistic gradient vector flow (PGVF) algorithm was used to track sciatic nerve region, obtaining an average Dice Similarity Coefficient (*DSC*) of 0.90. Alkhatib et al. [2], instead, proposed the adaptive median binary pattern (AMBP) as the texture feature of a tracking algorithm with an accuracy of 95%. Hadjerci et al. [9] proposed a segmentation pipeline including a pre-processing stage (filtering, de-noising, contrast enhancement), features extraction in a region of interest (ROI) and a support vector machine classifier. This method generated an average *DSC* of 0.81. However, even with good results, these approaches are parameter sensitive and require a certain degree of time-consuming manual intervention, especially for selecting the initial contour, thus possibly leading to segmentation errors.

After DL has emerged as leading machine learning tool in various research fields, including medical US analysis, recently some researches approached at the median nerve segmentation involving CNNs implementation. Hafiane et al. [11] combined a CNN, which detects the ROI around the nerve, with the PGVF method to delineate the median nerve contour on a dataset composed by US images extracted from 10 videos, each with 500 frames, from 10 patients. The results revealed an average *DSC* of 0.85.

In [16] the U-Net architecture [24] was used to identify the median nerve in the brachial plexus in US images, which were all pre-processed using linear Gabor binary patterns before being supplied to the U-Net for segmentation. They obtained an average *DSC* of 0.67, thus considering that the use of U-Net to directly segment the median nerve is not effective.

In [27], a multi-input similarity CNN was proposed to track the median nerve in US videos from 50 patients, which where asked to perform specific wrist motions. A total of 100 US videos of 6 s, each with 180 frames, were involved in this study, in which one target ROI containing the median nerve, manually defined in the first frame, is compared with candidate search images to find the more similar on the next frame of image stack. It’s worth to notice that this method relies on the manual identification of ROIs from expert clinicians as input to the model, which is a relevant limitation.

Hong et al. [15] proposed a fully DL framework based on U-Net for the localization and segmentation of the median nerve in US image sequences. The model, called DeepNerve, integrates also a MaskTrack [21], a video object segmentation technique, and a convolutional long short-term memory, LSTM [14], to process temporal information. Six patients were involved and a total of 24 videos, each with 420 frames and lasting 17.5 s. The images of the US sequences were cropped around the median nerve before being used to train and test the model. DeepNerve overcame segmentation performances of the conventional active contour model, generating an average *DSC* value of 0.897. Even though it currently reached the best outcomes, this method used images cropped around the median nerve as input, and the small number of patients involved limited the anatomical variability considered in the study.

Even in the very recent work by Festen et al. [6], two implementations of the U-Net model were considered on a dataset of 505 videos with 5560 annotated frames acquired involving 99 patients (with an average of 5.1 videos): one model was based on single-frame segmentation, the other was made using focus windows and spatial information from the previous segmented frame to redirect the focus of the search area for the next frame. Best results were achieved by the latter model with an average *DSC* of 0.88, but requiring the first frame manual definition by a user and ROIs as input to the model.

Despite the promising results, the main limitation of these DL methods is that they require the manual identification of a ROI around the median nerve, and this poses issues relevant to time consumption and inter-clinician variability.

At last, a very recent work conducted by Wu et al. [28] evaluated the performance in median nerve segmentation of different DL models, including DeepLabV3+, U-Net, FPN and Mask R-CNN [13], on US image sequences acquired from 36 subjects. The best performances were achieved by the Mask R-CNN with Intersection over Union (*IoU*) score close to 0.83. This work, however, focused on a small variety of anatomy and excludes unusual morphologies. In accordance with results achieved by Wu et al. [28], and in contrast with the other DL approaches found in the literature on this field, in which U-Net based models were chosen to face this task, we approached to the median nerve segmentation implementing a Mask R-CNN, which simultaneously detects target objects in the image and from that generates a high-quality segmentation mask for each instance. We aimed to provide a unified framework, which does not involve preliminary ROI identification or parameter-sensitive procedures.

In addition, our dataset is significantly different from the ones described in the DL state-of-art [6, 15, 16, 27, 28]: we focused on the morphology rather then the motion of the median nerve, thus considering US single frames instead of full frame sequences and involving in the study a greater number of patients, covering a higher anatomical variability. Table 1 summarizes the characteristics of these data sets and highlights the differences with our dataset.

Following sections present and discuss the proposed approach in details. The paper is organized as follows: in Section 2 our model is explained, the dataset used is defined and the experiments conducted are described; then, results are presented in Section 3 and discussed in Section 4; finally, in Section 5 the overall outcome and future perspective of this work are reported in conclusion of the paper.

**Table 1:** Overview of the US dataset characteristics in DL literature for median nerve segmentation, in terms of US acquisition site, dataset size (frames selection or frame sequences, total number of images) and patients involved in the study

	Acquisition site	Frame sequences	N. of US images	N. of patients
Kakade and Dumbali [16]	Brachial plexus forearm	No	11508	-
Wang et al. [27]	Carpal tunnel	Yes (100)	18000	50
Horng et al. [15]	Carpal tunnel	Yes (24)	10080	6
Festen et al. [6]	Proximal carpal tunnel inlet	Yes (505)	5560	99
Wu et al. [28]	Proximal carpal tunnel inlet	Yes (36)	18625	36
Proposed model	Proximal carpal tunnel inlet	No	246	103

## Materials and methods

In this study, we approached to the median nerve segmentation from transverse US images acquired at proximal carpal tunnel inlet deploying an end-to-end deep learning algorithm based on a Mask R-CNN implementation [13].

**Fig. 2:**
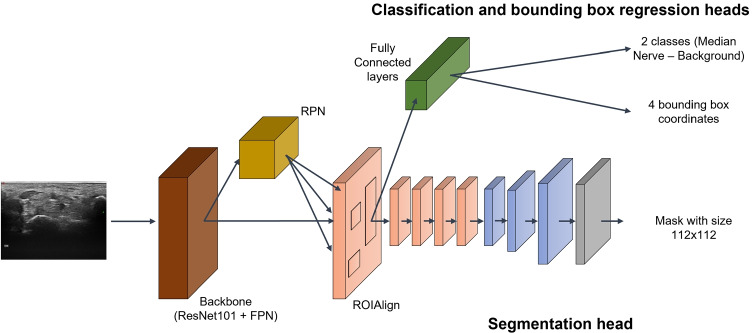
Schematic representation of model architecture, composed by a backbone, Region Proposal Network (RPN), and the three heads for classification, bounding box regression and segmentation, all fed from the ROIAlign with 100 ROI candidates. The segmentation head is represented more in details as it was provided with two additional transposed layers compared with original Mask-RCNN [13]

Mask R-CNN [13] is a CNN made of backbone, Region Proposal Network (RPN), ROIAlign and three heads, for classification, bounding box regression and segmentation. A schematic representation of the model proposed is shown in Fig. 2. As backbone we used the ResNet-101 [12] in combination with the Feature Pyramid Network (FPN) [18], allowing median nerve detection at multiple scales, which improves the performance of semantic segmentation over relying on a single scale analysis. As in the original implementation by He et al. [13], the RPN is used to generate proposals, i.e., rectangular regions in the US image with a high probability of containing the median nerve, which are predicted starting from anchors, which are here built with 5 different sizes and 3 different scales. The selected proposals are processed by the ROIAlign layer, which resizes the proposals to a constant *d* × *d* output matrix before feeding them to the heads.

The classification and regression heads are both made of two fully connected layers with 1024 neurons and an additional third fully connected layer, which has 2 neurons followed by a softmax function to predict the proposal class (i.e., median nerve or background) for the classification head and 4 neurons, linearly activated, to predicted the anchor box correction factors in the regression head. The segmentation head, instead, consists of four 3x3 convolutional layers with 256 filters, each activated with the rectified linear unit (ReLU), and three transposed convolutions with 256 2x2 filters, ReLU activated, which allow to recover spatial resolution up to 112x112. In this work, architectural changes from the original Mask R-CNN are introduced at the segmentation head to improve output mask resolution. In fact, the use of three transposed convolution layers instead of only one, as in the original Mask R-CNN, allows to increase the output resolution and deal with the fragmented and low-contrasted edges of the median nerve. To obtain stable convergence, the last layer performs a 1x1 convolution and it is activated by a sigmoid function.

The proposed method was developed based on the code in [1]; training and testing were performed using TensorFlow on a GPU GeForce RTX 2080. Dataset is not available due to ongoing research, but it could be provided upon request.

### Dataset

For this study, 103 patients with rheumatic and musculoskeletal disorders were recruited at the Rheumatology Unit of “Carlo Urbani” Hospital in Jesi (Ancona, Italy). All patients signed informed consent and the data acquisition was conducted in compliance with the Helsinki Declaration and with the approval of the local ethics committee (Comitato Etico Regione Marche, number 262). The US assessment was carried out using a MyLab Class C (Esaote SpA, Genoa, Italy) US system equipped with a 6–18 MHz linear probe taking transverse scans in accordance with the 2017 EULAR standardized procedures for US imaging in rheumatology [19]. US images at the proximal carpal tunnel inlet were acquired bilaterally from the patient wrists with the forearm resting supine on the examination bed and fingers in neutral position. The number of images per patient is variable, but of the same order of magnitude, and the few cases in which more than one image is acquired from the same patient were carefully considered as part of the same set (training or testing). Twenty-two out of 103 patients (21%) had a clinical diagnosis of CTS and some anatomical variants were observed and included in the study. The presence of the following anatomical variants was registered: bifid median nerve, persistent median artery, accessory muscles within the carpal tunnel. The images composing the dataset were acquired by three sonographers with different degrees of experience in musculoskeletal US (G.Sa.: 1 month with a dedicated intensive training; G.Sm.: 4 years; E.Fi.: more than 20 years of experience). Images considered of insufficient quality were excluded from the dataset after a revision made by the expert sonographers. Manual annotation was performed by one sonographer (G.Sa.) under the supervision of the other two.

The annotations were used as ground truth for the training of the CNN proposed for the segmentation task. The dataset included a total of 246 US images with size equal to 606x468 pixels. The images with the corresponding masks were resized to 512x512 pixels using bilinear interpolation. In addition, the images were zero-padded at right-most and bottom-most edges to get squared images with a size multiple of 32, as required by the FPN, while keeping the aspect ratio unchanged.

### Experimental setup

The dataset was randomly split by patients, whose demographic and clinical characteristics matched inclusion criteria designed by rheumatologists prior performing the acquisition. To cope with the small amount of data available 5-fold cross validation was performed. All ablation studies and comparison with state of the art models were conducted training in 5-fold cross validation and testing on model with best validation loss.

Considering the relatively small size of our dataset and to reduce the chances of overfitting, during training data augmentation was performed on-the-fly by randomly scaling of 80*%* to 120*%* of original size and translating of − 20*%* to 20*%* on both x- and y-axis independently, and performing random rotation between (-10^∘^, 10^∘^) and shearing between (− 2^∘^, 2^∘^). We ensure to consider ranges for the affine transformations for which the nerve remains always visible in the images.

To improve training speed and accuracy, we performed transfer learning initializing all layers of the model except for the input layers of the network heads with weights computed on the COCO dataset [17]. Freezing the backbone while focusing on network heads training aimed to increase features extraction process through the support of a large natural images dataset.

The training was performed following guidelines for training CNNs, including dropout and weight decay as regularizer. Stochastic Gradient Descent was deployed as optimizer for 150 epochs with a learning rate of 0.001 and momentum of 0.9. A total of 256 anchors per image was used, with varying size (32, 64, 128, 256 and 512) and aspect ratios (1:1, 2:1, 1:2). These values were chosen considering the median nerve section dimension. The ROIAlign resized proposals to a fixed output size of 14x14 considering a total of 100 training ROIs per image, as a trade off between accuracy and memory consumption.

The network was trained defining a multi-task cross-entropy loss on each ROI combining the loss of classification, localization, and segmentation mask equally weighted: *L* = *αL*_*cls*_ + *βL*_*bbox*_ + *γL*_*mask*_, where *L*_*cls*_ and *L*_*bbox*_ are class and bounding box losses of Faster R-CNN, respectively, and *L*_*mask*_ is the mask loss defined in [23], and *α*, *β* and *γ* are constants, which we set to 1 after experimental investigations.

In addition, from the median nerve segmentation obtained the CSA was calculated knowing that a single pixel in the US images of our dataset has each dimension equal to 0.062*mm* × 0.062*mm*. The CSA was calculated only on *TP* predictions.

### Comparison with literature and ablation studies

As mentioned in Section 1.1, a relatively small number of studies is focused on DL application on US for CTS assessment and in most of these contributes, as in [16], [15] and [6], U-Net models were chosen to get the median nerve segmentation. Hence, even though our dataset is composed by still US images instead of US videos as in current literature, and thus these works are not superimposable, we conducted a performance comparison among our model and some U-Net based approaches. In this way, we want to prove the effectiveness of the deployment of a Mask R-CNN architecture rather than U-Net models to obtain an end-to-end framework, which accurately segments the median nerve without the requirement of any a priori localization or parameter-sensitive post-processing.

We considered the architectures deployed in [15] of the U-Net, which kept the original implementation on this state-of-art network from [24], and a Lightweight U-Net, in which the network’s depth was reduced from 5 to 4 layers and batch normalization was used as a follow-up step to the first convolution in each layer to avoid premature convergence. To evaluate the best performances of these models in comparison with the proposed one, we trained them using the Binary Cross-Entropy (*BCE*) loss, which is the default loss for segmentation models, and also combining the *BCE* loss with the *DSC* loss (*BCE* − *DSC* loss), expected to provide more stability to the models [8]. The *DSC* is also the metric mainly used to judge model performance in terms of segmentation, that was calculated in this work as in Eq. (1):

1 $$DSC=\frac{2 \times \mid A_{gt}\cap A_{mask}\mid}{\mid A_{gt}\mid + \mid A_{mask}\mid}$$

where *A*_*gt*_ and *A*_*mask*_ are the ground truth and predicted segmentation areas, respectively.

As ablation study we investigated if a larger backbone network yields higher accuracy: thus, we compared the Resnet-101 combined with FPN (ResNet-101-FPN) with the ResNet-50-FPN. To evaluate if augmentation leads to a greater generalization of the model, we included in the ablation studies an experiment training the model without any type of augmentation. In addition, we evaluated the effect of having a different number of transposed convolutions in the segmentation head. This was done to assess the effects of an increased resolution of the output of the segmentation head on the overall segmentation performance. The segmentation head was tested with one (Mask28) and two (Mask56) transposed convolutional layers, leading to the output size of the head of 28x28 and 56x56, respectively. For a fair comparison, the ablation studies were performed using fivefold cross validation, same training settings and computational hardware.

### Performance metrics

Precision (*Prec*), Recall (*Rec*) and Mean Average Precision (*mAP*) are used to evaluate the performance in median nerve localization. Precision (*Prec*) and Recall (*Rec*) were computed as indicated by Eq. (2) and Eq. (3), respectively:

2 $$Prec = \frac{TP}{TP + FP}$$

3 $$Rec = \frac{TP}{TP + FN}$$

where *TP*, *FP* and *FN* denote the number of true positives, false positives and false negatives, respectively. We considered a *TP* prediction if the detected bounding box overlapped the bounding box surrounding the ground truth segmentation for at least 70% and had confidence higher than 0.98. We considered a wrong positive detection as *FP*, in which the predicted bounding box didn’t reach the 70% of overlapping threshold with the ground truth bounding box. We considered a *FN* when the actual instance was not detected, thus no bounding box was predicted at all. The value of 70% as threshold for defining *TP*, *FP* and *FN* has been chosen to provide more strict and reliable segmentation from nerve detection: we considered the standard Pascal VOC evaluation practice [5] with minimum overlapping at 50% between predicted and ground truth bounding boxes as not accurate enough for properly measure CSA, fundamental parameter for CTS diagnosis. Mean Average Precision (*mAP*), which represents the average of the area under the Recall-Precision curve, was also computed. The median nerve segmentation performance was measured using the *DSC* as defined in Eq. (1).

In addition, the CSA was automatically calculated from the median nerve section predicted by the algorithm, knowing the dimensions of a single pixel (0.062*mm* × 0.062*mm*) in the US images. The CSA was calculated only on *TP* predictions and compared with manual measurements performed by the sonographers measuring the mean absolute error (*MAE*).

### Statistical analysis

We assessed if the data were normally distributed by using Kolmogorov-Smirnov test, using an *α* value of 0.05. As the data are non-normally distributed (the p-value of Kolmogorov-Smirnov test is equal to 0.048e-143), we performed a Mann Whitney test with *α* = 0.05 to compare the CSA measurements.

The agreement in the CSA measurements between the sonographer annotation (i.e., the gold standard) and the algorithm was calculated using a two-way mixed-effects intra-class correlation coefficient (ICC) with 95% confidence interval (CI). The ICC is regarded as excellent if above 0.9, as good if between 0.75 and 0.9.

The statistical tests were performed using Python and Scipy library.

## Results

The proposed model achieved good performances both in detection and segmentation of median nerve section: we obtained on average *mAP*, *Rec*, *Prec* and *DSC* equal to 0.936 ± 0.235, 0.938 ± 0.233, 0.916 ± 0.245 and 0.868 ± 0.201, respectively. The average inference time for each image on a GPU GeForce RTX 2080 was 1.7 s, which could be further improved with more powerful computational resources.

**Table 2:** Performance evaluation metrics in terms of mean value and standard deviation. Mean average precision (*mAP*), Recall (*Rec*), Precision (*Prec*) and Dice Similarity Coefficient (*DSC*) are reported for the proposed model and the ablation studies conducted over it: Mask-R50 is the model trained using as backbone Resnet50 combined with FPN; NoAug is the model trained using no augmentations on the training data; Mask28 and Mask56 are variants of the model with a different output resolution from the segmentation head, including one and two transposed convolutional layers, respectively

	*mAP*	*Rec*	*Prec*	*DSC*
Mask-R50	0.889 ± 0.277	0.888 ± 0.271	0.862 ± 0.261	0.843 ± 0.208
NoAug	0.891 ± 0.241	0.902 ± 0.294	0.870 ± 0.308	0.838 ± 0.247
Mask28	0.908 ± 0.364	0.923 ± 0.254	0.877 ± 0.285	0.821 ± 0.261
Mask56	0.926 ± 0.235	0.895 ± 0.284	0.899 ± 0.270	0.843 ± 0.219
Proposed Model	0.936 ± 0.235	0.938 ± 0.233	0.916 ± 0.245	0.868 ± 0.201

Table 2 summarizes the results obtained modifying the model architecture by using a different backbone (Mask-R50) and considering two different output resolution of the segmentation head, leading to masks with size 28x28 (Mask28) and 56x56 (Mask56).

**Table 3:** Comparison of segmentation performance in terms of *DSC* of the proposed model and of the U-Net and Lightweight U-Net trained using two different losses, i.e., the *BCE* loss and the *BCE* − *DSC* loss

	DSC
U-NET (*BCE* loss)	0.783 ± 0.229
U-NET (*BCE* − *DSC* loss)	0.822 ± 0.205
Lightweight U-NET (*BCE* loss)	0.780 ± 0.195
Lightweight U-NET (*BCE* − *DSC* loss)	0.764 ± 0.216
Proposed Model	0.868 ± 0.201

**Fig. 3:**
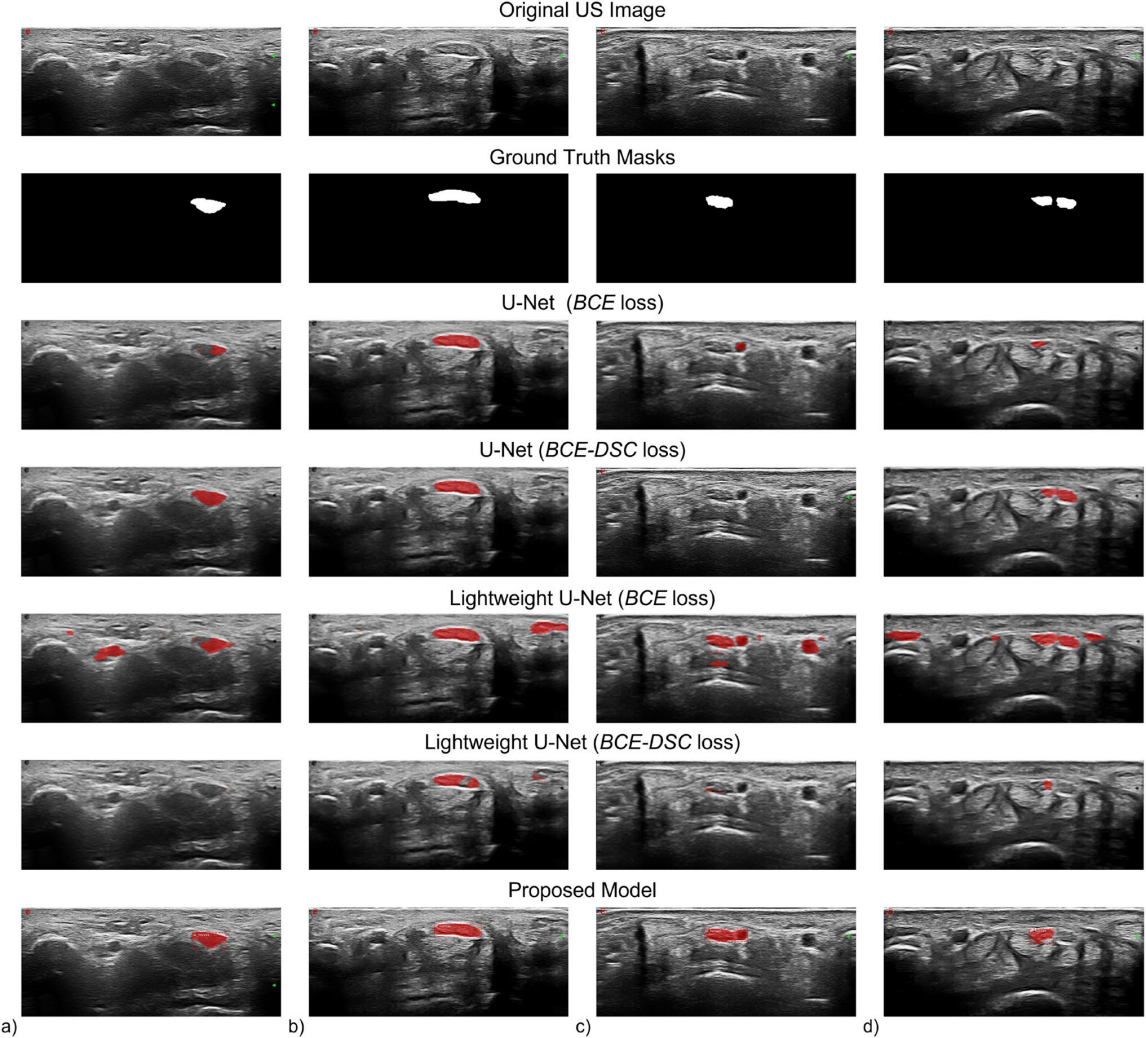
Four visual samples of the median nerve section. From top to bottom row: original US image, ground truth mask, U-Net trained with *BCE* loss prediction, U-Net trained with *BCE* − *DSC* loss prediction, Lightweight U-Net trained with *BCE* loss prediction, Lightweight U-Net trained with *BCE* − *DSC* loss prediction, proposed model prediction. For displaying purpose, only the upper part of the US images, which contains the median nerve section, is shown

To evaluate the segmentation capability, we compared the proposed model with the U-Net and Lightweight U-Net models deployed in literature, referring in particular to [15]. Table 3 outlines the segmentation performances of these models in term of *DSC*, expressed as mean ± standard deviation value. Visual samples are shown in Fig. 3: sample of a healthy median nerve section (Fig. 3a), sample acquired from a patient with CTS (Fig. 3b), sample containing a prominent persistent median artery (Fig. 3c) and a sample of a bifid median nerve (Fig. 3d). Moreover, the *CSA* was measured on the predicted median nerve sections. Without considering *FP* and *FN* predictions, the values were comparable with the ones manually measured by the sonographer with a *MAE* of 0.918 *mm*^2^. On average, *CSA* measured by the sonographer was 10.360 ± 4.520 *mm*^2^, while *CSA* automatically calculated from the predicted segmentation masks was 10.380 ± 4.240 *mm*^2^, with no significant difference (p = 0.88). The agreement between the automatic algorithm measurement and the sonographer manual measurement of the CSA is remarkable [ICC 0.97 (95% CI 0.94–0.98)].

## Discussion

Despite the increasing interest in US support for CTS assessment and the well-established usefulness as confirmatory diagnostic test of the median nerve size measurement, US imaging is still struggling to be regularly employed in diagnostic work-up. This is partially due to the high competence required to perform and interpret US at carpal tunnel level, the lack of protocols standardization and the high variability among sonographers’ evaluations. Therefore, in this work we proposed an end-to-end DL approach to support sonographers for median nerve compression evaluation. Specifically, we approached the median nerve segmentation directly from US developing a Mask R-CNN model, obtaining remarkable results for both localization (*mAP* = 0.936 ± 0.235, *Rec* = 0.938 ± 0.233, *Prec* = 0.916 ± 0.245) and segmentation (*DSC* = 0.868 ± 0.201). Moreover, the automatic measurement of the *CSA* from the predicted median nerve section resulted to be in agreement with the manual measurement of the *CSA* (with an average *MAE* of 0.918 *mm*^2^), implying the possibility to reduce reliance on sonographer expertise in carpal tunnel US evaluation while increasing intra- and inter-observer reliability.

Differently to other semantic segmentation models, Mask R-CNN solves the segmentation problem on top of localization, producing a mask for each recognized object, instead of just one final mask, thus leading to more accurate results. Previous works, in fact, approached the problem deploying U-Net based models [6, 15, 16], but they all involved some manual intervention in ROI identification or nerve contour definition to obtain good median nerve segmentation. The most similar work from a methodological point of view is the one from [28], in which the best results are achieved implementing a Mask R-CNN model; however, even with less data, we achieved higher performance on our dataset, which includes a greater number of patients and thus a higher variability, confirming the instance segmentation as more suitable and better performing than semantic segmentation approaches.

Therefore, we compared our model with different implementation of U-Net models proving the better outcomes reached by our model as evidenced by the *DSC* values reported in Table 3. In addition, Fig. 3 shows some representative samples of the region of the median nerve from predictions of the proposed model and of the U-Net based models. The U-net models often confounded the median nerve section with other rounded structures regardless their shape or characteristic pattern. The Lightweight U-Net models, in particular, obtained the worst performances generating a lot of *FP* predictions, thus resulting not very effective in median nerve localization. Our model, instead, incorrectly identifies only the infrequent morphologies, thus all images belonging to the same patients which present a rare anatomical variants at carpal tunnel level.

In few cases, though, our Mask R-CNN didn’t lead to a perfect segmentation, but even in such cases it achieved better performances than the other models. As displayed in Fig. 3, the model struggles to interpret US images with relatively infrequent anatomical variants, like in contiguity with vessels as in Fig. 3c, and in presence of bifid median nerve as in Fig. 3d.

In addition, poor definition of nerve borders, presence of multiple rounded hypoechoic areas, complex fascicular pattern typical of peripheral nerves and inhomogeneities of the nerve section could contribute to make the detection harder. Results of the ablation studies reported in Table 2 highlighted how a deeper backbone granted good outcomes, and it could be appreciated that concatenation of several augmentations provides better results and more generalization than considering no augmentations on training data. In the future, we could also consider introducing augmentations on color, like brightness variation. As in Table 2, the increase in the output mask resolution from the segmentation head provided generally more accurate results. In fact, there are considerable improvements passing from 28x28 to 112x112 pixels output mask resolution, and lower performances are also visible in Mask56 compared to proposed model. In addition, in Table 2 we could appreciate that concatenation of several augmentation generalized results better than considering single operations, like only rotation and only translation, on training data. In the future, we could also consider introducing augmentation on color, like brightness variation.

To increase the algorithm generalization, indeed, it is fundamental to expand the dataset with US images encompassing a wider spectrum of normal anatomy at carpal tunnel level.

In future work, it could be interesting to consider pretraining on larger US existing datasets to improve model accuracy and reliability. The dataset should also be enlarged considering different US image acquisition equipment, lower-frequency probes and maybe involving more research centers in the study to strengthen generalizability further. It could be interesting even to approach the problem including different diagnostic tests and imaging the median nerve at the carpal tunnel from a different perspective and considering different wrist motion.

## Conclusion

In this work, we proposed a DL approach that proved to be a reliable tool for the automatic segmentation of the median nerve in US images reaching a mean *DSC* equal to 0.868 ± 0.201, from which directly measure the CSA of the median nerve. Even though improvements need to be done to be deployed in the clinical practice, the promising results obtained have shown the potentiality of such DL approach, which could allow to support beginner sonographers, to introduce standardized protocols and thus to possibly support CTS diagnosis through US inspection.

In future, spatio-temporal information [3] should be included: other than improving median nerve segmentation, US videos allow also to evaluate an additional relevant parameter for CTS, the median nerve mobility. Distance-field regression for accurate nerve delineation could be investigated, too, considering the promising results achieved in close fields [7]; and alternatively, improving the detector of a Cascade Mask R-CNN as in [30] could be explored to minimize inaccurate localization and low recognition accuracy.
